# Changes in attentional resources during the acquisition of laparoscopic surgical skills

**DOI:** 10.1093/bjsopen/zraa012

**Published:** 2020-12-23

**Authors:** M Thomaschewski, M Heldmann, J C Uter, D Varbelow, T F Münte, T Keck

**Affiliations:** Department of Surgery, University of Lübeck, Lübeck, Germany; Department of Neurology, University of Lübeck, Lübeck, Germany; Institute of Psychology II, University of Lübeck, Lübeck, Germany; Department of Neurology, University of Lübeck, Lübeck, Germany; Department of Surgery, University of Lübeck, Lübeck, Germany; Department of Neurology, University of Lübeck, Lübeck, Germany; Institute of Psychology II, University of Lübeck, Lübeck, Germany; Department of Surgery, University of Lübeck, Lübeck, Germany

## Abstract

**Background:**

Increasing familiarity and practice might free up mental resources during laparoscopic surgical skills training. The aim of the study was to track changes in mental resource allocation during acquisition of laparoscopic surgical skills.

**Methods:**

Medical students with no previous experience in laparoscopic surgery took part in a 5-week laparoscopic training curriculum. At the beginning and end of the training period, one of the training tasks was combined with a secondary auditory detection task that required pressing a foot switch for defined target tones, creating a dual-task situation. During execution of the two concurrent tasks, continuous electroencephalographic measurements were made, with special attention to the P300 component, an index of mental resources. Accuracy and reaction times of the secondary task were determined.

**Results:**

All 14 participants successfully completed the training curriculum. Target times for successful completion of individual tasks decreased significantly during training sessions (*P*  <0.001 for all tasks). Comparing results before and after training showed a significant decrease in event-related brain potential amplitude at the parietal electrode cluster (P300 component, W = 67, *P* = 0.026), but there were no differences in accuracy (percentage correct responses: W = 48, *P* = 0.518) or reaction times (W = 42, *P* = 0.850) in the auditory detection task.

**Conclusion:**

The P300 decrease in the secondary task over training demonstrated a shift of mental resources to the primary task: the surgical exercise. This indicates that, with more practice, mental resources are freed up for additional tasks.

## Introduction

The rapid progress in surgery with increasing use of laparoscopic and robotic-based minimally invasive techniques presents new challenges for the acquisition of surgical skills. Simulation-based training is used widely to develop motor skill learning^1^ needed for minimally invasive surgery (MIS) (similar to that when learning a musical instrument^2^^,^^3^). The learner must cope with different haptics, two-dimensional image interpretation, and the fulcrum effect with paradoxical movement in many situations. Attentional resources available in novices are limited owing to the complexity of the task, especially at the beginning of the learning curve, interfering with ability to cope with disturbances or complications during the exercise or during an operation. A number of different simulation environments have been developed to study the learning trajectories for different skills required for MIS, and to assess the accuracy, speed, and efficiency of movements^1^^,^^4–9^.

The neuroscience literature on the acquisition of motor skills is sparse regarding the neural underpinnings and prerequisites of skill learning^10–13^, and this has been largely neglected in the development of simulator-based MIS training programmes. Instead, the number of repetitions has been used widely as rough discriminator of motor skill competence.

Brain event-related brain potentials (ERPs) are small voltage fluctuations in the human electroencephalogram (EEG) that can be recorded non-invasively from the intact scalp of a volunteer, and are induced by external events, such as sensory stimuli^14^. The ERPs are extracted from the EEG by averaging over a number of similar events.

The execution of two (or more) concurrent tasks (dual tasking) is integral to cognitive performance, and requires attentional control to allocate resources to each of the tasks^15^^,^^16^. As an example, in conditions with diminished mental resources, such as Alzheimer’s disease, people stop walking when they start to talk, because the mental resources are taken up by talking and are not sufficient to support parallel walking^17^. Inability to perform dual tasking during an operation is commonly encountered among novice surgical trainees^18^^,^^19^, but may also be seen in experienced surgeons during complex operations.

ERPs have been used extensively^20–25^ to assess mental resource allocation in dual-task situations. In particular, the P300 component of the ERP to stimuli in the secondary task has been used as a marker to track control dynamics in the primary task^23^^,^^26–28^.

This experimental study involved a dual-task situation in which an additional auditory detection task was included during a laparoscopic surgical task. Mental resources at the beginning and end of a 5-week laparoscopic training programme were evaluated using ERPs. The hypothesis was that laparoscopic training and increasing familiarity in performing laparoscopic tasks should free up mental resources for additional (secondary) tasks.

## Methods

### Lübeck Toolbox curriculum

**Fig. 1:**
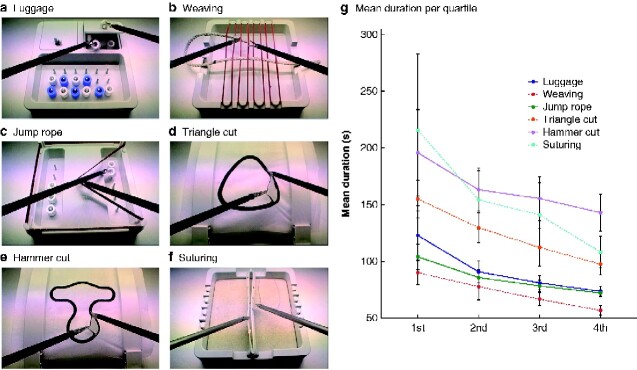
Six training tasks of the Lübeck Toolbox and duration of exercises Tasks: **a** pack your luggage, **b** weaving, **c** Chinese jump rope, **d** triangle cut, **e** hammer cut, and **f** suturing. **g** Mean(s.d.) duration per quartile of performed runs per exercise.

The Lübeck Toolbox (LTB) curriculum was used for MIS training. It consisted of six related exercises: pack your luggage, weaving, Chinese jump rope, triangle cut, hammer cut, and suturing (*Fig. 1*). The LTB exercises were performed as described previously^9^.

The LTB video box trainer (LTB Germany, Lübeck, Germany) and the following instruments were used for MIS training: atraumatic grasping forceps (Endopath^®^ 5-mm Overholt 5DCD; Ethicon Endo-Surgery, Cincinnati, Ohio, USA), laparoscopic scissors (Endopath^®^ laparoscopic scissors, curved, 5mm; Ethicon Endo-Surgery) and needle drivers (Endopath^**^®^**^ 5-mm needle holder E705R; Ethicon Endo-Surgery). Vicryl^**™**^ SH PLUS 3-0 (Ethicon, Norderstedt, Germany) was used as suture.

### Lübeck Toolbox curriculum analysis

The number of repetitions and duration of each repetition to reach a defined training criterion was used to operationalize the learning progress. These numbers were obtained from previous studies on the LTB curriculum^8^^,^^9^. For each exercise and subject, quartiles of the numbers of repetitions were calculated and used to derive the mean duration per quartile. For each exercise, the Friedmann test was applied to test for significant learning progress (R version 3.5.1; R Foundation for Statistical Computing, Vienna, Austria).

### Study design and event-related potential task

This was an experimental study performed in the Departments of Surgery and Neurology, University of Lübeck, Germany. The study was approved by the Ethics Committee of the Universität zu Lübeck (ethics committee protocol 19-095). Informed consent was obtained from all participants at the beginning of the study. Medical students with no previous experience in laparoscopic surgery took part in a 5-week laparoscopic training programme according to the LTB curriculum. At the beginning and the end of the LTB curriculum, the study participants participated in a dual task with EEG recording. The primary task was the pack-your-luggage exercise from the LTB, and the secondary task comprised an auditory change detection task. A jingle at the beginning of each run indicated ‘begin with the pack-your-luggage exercise’. With an offset of 2 s, the auditory change detection task started. Two alternating tones (800 and 880 Hz, interstimulus interval (ISI) 1100 ms) were presented via headphones. With a probability of 0.1, a tone was repeated instead of presenting an alternative one. It was the subject’s task to indicate a sound repetition by pushing a foot switch with their dominant foot. Sound loudness was adjusted individually at the beginning of each session to a comfortable level. The change detection task was implemented in Psychtoolbox version 3.0.14 (http://psychtoolbox.org, running under Matlab^®^ 2018b (https://mathworks.com), and Windows^®^ 7 (Microsoft, Redmond, Washington, USA)). The tones were presented via Sennheiser headphones (Wedemark, Germany) connected to a Steinberg U12 USB audio interface (Hamburg, Germany). Auditory stimulus presentation was stopped when participants finished the training task.

### Electroencephalogram recording and analysis

EEGs were recorded using 16 gtec Sahara dry electrodes connected to a gtec USB amplifier system (Thanstetten, Austria). Electrodes (F3, Fz, F4, Fc5, Fcz, Fc6, C3, Cz, C4, Cp5, Cp6, P3, Pz, Oz) were mounted in an elastic cap and referenced to the right mastoid. To control for eye movement artefacts, bipolar standard electrodes were placed at the left and right outer canthus of the eyes (horizontal electro-oculographic (HEOG) aretefacts), and below and above the right eye (vertical electro-oculographic (VEOG) artefacts). Data were recorded using a sampling rate of 250 Hz (bandpass filtered 0.1–30 Hz). EEGLAB^39^ and ERPLAB^40^ toolboxes were used for EEG data analysis. The EEG data processing pipeline comprised epoching to the stimulus presentation (–1500 to 1500 ms), and identification of EOG and other artefacts using independent component analysis implemented in EEGLAB. Subsequently, identified artefact components were subtracted from the EEG data, and individual ERPs for the conditions before/after training × standard/target were calculated by averaging per condition and subject using the mean activity between –100 and 0 ms as baseline. Finally, ERPs were individually standardized using z-transformation. Because of excessive movement artefacts, two subjects had to be excluded from the analysis, resulting in a final sample of 12 subjects. Parietal attention effects were parameterized by averaging potentials across the centroparietal/parietal electrode positions Cp5, Cp6, P3, Pz, and P4, and calculating the mean amplitude between 400 and 600 ms after stimulus presentation. For the statistical evaluation of the training effect, we first calculated the difference between standard and target tone per subject and condition (before/after training). The resulting difference scores were subjected to a Wilcoxon matched pairs test comparing the before with the after training condition. The use of the non-parametric Wilcoxon matched-pairs test is determined by the group size.

## Results

### Lübeck Toolbox curriculum

All 14 participants (mean(s.d.) age 26.07(2.33) years; 10 women) successfully completed the LTB curriculum. The median number of repetitions participants needed to reach the criterion for the LTB exercises was 30 (range 24–70) for the luggage task, 40 (24–72) for weaving, 32 (12–71) for Chinese jump rope, 27 (11–76) for triangle cut, 23 (9–52) for hammer cut, and 17 (11–56) for suturing. The mean exercise times for the LTB tasks per quartile decreased significantly over time for each of the seven training sessions all (*P* < 0.001) (*Fig. 1*).

### Auditory task: event-related potentials and behaviour

**Fig. 2:**
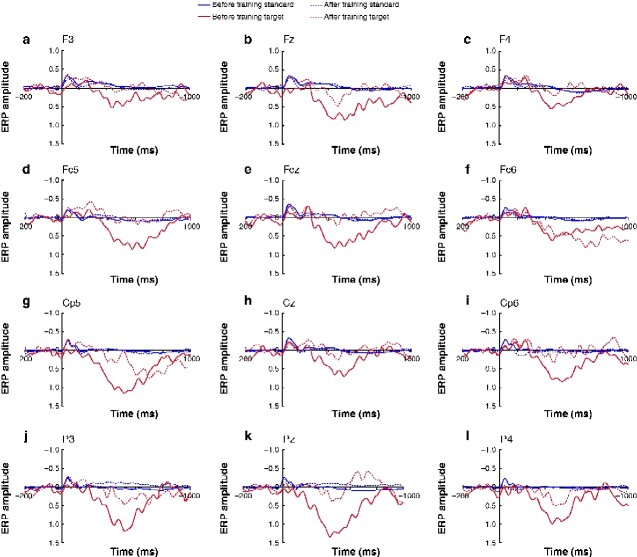
Grand average of z-standardized event-related potentials obtained time-locked to presentation of the standard alternating tone and repeating target tone Recordings from electrode positions **a** F3, **b** Fz, **c** F4, **d** Fc5, **e** Fcz, **f** Fc6, **g** Cp5, **h** Cz, **i** Cp6, **j** P3, **k** Pz, and **l** P4. Target tones were associated with a large parietal P300 component at the beginning of the training period which was greatly diminished in the session at the end of training. Baseline was –100 to 0 ms. ERP, event-related potential.

**Fig. 3:**
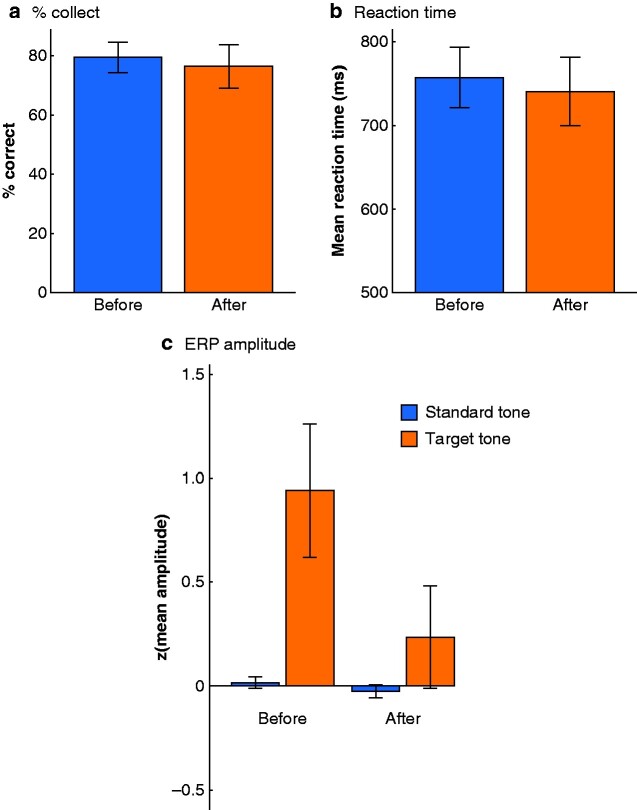
Results for auditory change detection task, and amplitude of z-transformed event-related potentials Mean(s.d.) **a** percentage correct and **b** reaction times for auditory change detection task before and after training. **c** Mean(s.d.) amplitude of z-transformed event-related potentials (ERPs) averaged across electrode positions Cp5, Cp6, P3, Pz, and P4 before and after training.

ERP analysis revealed a large positivity at central–parietal electrode sites for the target tone before training (*Figs 2* and *3*). This effect was absent after training. Comparison of the difference target–standard tone between before and after training revealed a significant effect at the parietal electrode cluster (W = 67, *P* = 0.026). This decrease in ERP amplitude was not accompanied by significant before–after differences in accuracy (percentage correct responses: W = 48, *P* = 0.518) and reaction times (W = 42, *P* = 0.850) in the auditory change detection task.

## Discussion

The LTB features several tasks that mimic surgical techniques used in minimally invasive laparoscopic surgery. The present study corroborated earlier results obtained with the LTB^8^^,^^9^, that naive trainees rapidly improved performance to criterion within several training sessions. The repeated (target) tones were associated with a P300 component in the ERP.

The amplitude of the P300 component has been interpreted as an index for mental resources that are available for processing of the task at hand, with reduced P300 indicating reduced mental resources^23^^,^^25–28^. The reduced P300 amplitude to auditory target stimuli at the end of the training period therefore indicates that fewer resources are allotted to the secondary auditory task and, by inference, more resources are available for the primary surgical motor task. The increasing speed and precision of the surgical task was thus achieved by recruitment of mental processing sources, shielding the important primary task from interference by the auditory task. It seems reasonable to conclude that, in this early training stage, improvement is mainly due to reallocation of mental resources.

In both animals and humans, learning of a new skill entails an initial phase of rapid improvement in performance, and a subsequent phase of more gradual improvements as skills become more automatic^29–31^.These stages are characterized by distinct neural mechanisms. In experimental studies, the early stages of visuomotor learning have been shown to involve the dorsomedial striatum, equivalent to the caudate nucleus in primates, whereas the later phases concerning automatization of the behaviour are supported by the dorsolateral striatum, equivalent to the putamen in primates^29^^,^^32^^,^^33^. Functional neuroimaging has been used to describe distinct early and late phases of motor sequence learning with regard to activation of the motor cortex in adult humans^31^. It has also been shown that, with extensive practice, dual-task costs diminish and task coordination improves owing to task automatization^34^^,^^35^, and/or learning of the component tasks with practice^36–38^.

The present study attests to the value of ERPs in tracking changes in mental resource allocation during acquisition of MIS skills. Neuroscientific measures can reveal different stages of skill learning, and have the potential for evaluation of MIS surgical skills acquisition independent of roughly defined numerical repetitions. Although the present research was restricted to novice learners and only tracked the first stages of skill learning, studies examining dual-task performance over a long period of time as well as those involving expert surgeons might point to greater applicability, such as the ability to differentiate between trained and untrained surgeons or indicators of progress during a learning curve.
